# Non-invasive Cardiac Output Monitoring and Assessment of Fluid Responsiveness in Children With Shock in the Emergency Department

**DOI:** 10.3389/fped.2022.857106

**Published:** 2022-04-07

**Authors:** Pranali Awadhare, Radha Patel, Tracy McCallin, Kiran Mainali, Kelly Jackson, Hannah Starke, Utpal Bhalala

**Affiliations:** ^1^The Children’s Hospital of San Antonio, San Antonio, TX, United States; ^2^University of the Incarnate Word School of Osteopathic Medicine, San Antonio, TX, United States; ^3^Department of Pediatrics, University Hospitals Rainbow Babies and Children’s Hospital, Cleveland, OH, United States; ^4^Driscoll Children’s Hospital, Corpus Christi, TX, United States; ^5^Department of Pediatrics, Texas A&M University, College Station, TX, United States; ^6^Department of Anesthesiology and Critical Care Medicine, University of Texas Medical Branch, Galveston, TX, United States

**Keywords:** fluid responsiveness, shock, children, hemodynamic monitoring, electrical cardiometry

## Abstract

**Introduction:**

The assessment of fluid responsiveness is important in the management of shock but conventional methods of assessing fluid responsiveness are often inaccurate. Our study aims to evaluate changes in objective hemodynamic parameters as measured using electrical cardiometry (ICON^®^ monitor) following the fluid bolus in children presenting with shock and to evaluate whether any specific hemodynamic parameter can best predict fluid responsiveness among children with shock.

**Materials and Methods:**

We conducted a prospective observational study in children presenting with shock to our emergency department between June 2020 and March 2021. We collected the parameters such as heart rate (HR), respiratory rate (RR), systolic blood pressure (SBP), diastolic blood pressure (DBP), mean arterial pressure (MAP), and hemodynamic data such as cardiac output CO), cardiac index (CI), index of contractility (ICON), stroke volume (SV), stroke index (SI), corrected flow time (FTC), systolic time ratio (STR), variation of index of contractility (VIC), stroke volume variation (SVV), systemic vascular resistance (SVR), and thoracic fluid content (TFC) using the ICON monitor before and after fluid bolus (FB). We assessed percent change (Δ) and used paired-sample Student’s *t*-test to compare pre- and post-hemodynamic data and Mann–Whitney *U*-test to compare fluid responders and non-responders. *P*-Values < 0.05 were considered statistically significant.

**Results:**

We recorded 42 fluid interventions in 40 patients during our study period. The median IQR age was 10.56 (4.8, 14.8) years with male/female ratio (1.2:1). There was a significant decrease in ΔRR [−1.61 (−14.8, 0); *p* = 0.012], ΔDBP [−5.5 (−14.4, 8); *p* = 0.027], ΔMAP [−2.2 (−11, 2); *p* = 0.018], ΔSVR [−5.8 (−20, 5.2); *p* = 0.025], and ΔSTR [−8.39 (−21, 3); *p* = 0.001] and significant increase in ΔTFC [6.2 (3.5, 11.4); *p* = 0.01] following FB. We defined fluid responders by an increase in SV by ≥10% after a single FB of 20 ml/kg crystalloid. Receiver operating curve analysis revealed that among all the parameters, 15% change in ICON had an excellent AUC (0.85) for the fluid responsiveness.

**Conclusion:**

Our study showed significant changes in objective hemodynamic parameters, such as SVR, STR, and TFC following FB in children presenting with shock. A 15% change in ICON had an excellent predictive performance for the fluid responsiveness among our cohort of pediatric shock.

## Introduction

Shock is a leading cause of morbidity and mortality in the pediatric patients worldwide (1, 2). The prevalence of sepsis and septic shock has been reported to be around 1–26% of shock cases with mortality rates ranging from 5 to 35% in hospitalized children globally (3, 4). Appropriate fluid resuscitation is crucial in the management of children with shock (5). The current American College of Critical Care Medicine (ACCM), Pediatric Advanced Life Support (PALS), and Surviving Sepsis Campaign Guidelines have focused on the implementation of early and goal-directed fluid therapy (6, 7). Many studies have shown that mortality in pediatric patients with septic shock has been significantly decreased with aggressive fluid administration (8, 9). However, overzealous fluid administration can also lead to fluid overload (FO) and has been associated with complications such as acute respiratory distress syndrome (ARDS), which results in poor outcomes including increased hospital length of stay and mechanical ventilator days (10–13). As a result, in the recent decades, a more restrictive approach for fluid resuscitation has emerged in adults and children vs. the usual aggressive fluid therapy (14–16).

Despite ongoing extensive research related to fluid management in septic shock, the optimal amount of fluid to administer in early resuscitation of pediatric shock remains uncertain (17). Therefore, it is imperative to assess the clinical and hemodynamic responses before and after each fluid bolus (FB) to guide resuscitation and to determine the presence or absence of FO. Traditional use of subjective findings such as pulse volume, capillary refill time, and clinical signs of hydration status to predict fluid responsiveness (FR) has been proven to be unreliable (18, 19). In the recent decades, objective hemodynamic parameters have gained popularity and have been shown to reliably predict FR in adults (20, 21). While there is a growing body of the literature on the use of non-invasive devices for objective hemodynamic monitoring, there is a paucity of the literature related to the assessment of FR using these measures in children with shock (22).

ICON monitor, which is based on a novel technology of electrical cardiometry (EC), is one such non-invasive hemodynamic monitoring device and has been studied for the assessment of FR in adult patients with the promising results (23, 24). EC technique uses signals generated by the surface electrodes to measure the alterations in thoracic impedance. The changes in bio-impedance to the flow of erythrocytes in the aorta are computed into an algorithm allowing continuous hemodynamic monitoring (25). In our pilot study, we aim to determine changes in subjective and objective measures of hemodynamic status before and after FB in children with shock using ICON monitor. We also sought to assess whether changes in objective parameters could predict FR in these children.

## Materials and Methods

### Study Design and Selection of Participants

We conducted a prospective observational pilot study in children presenting with shock to our emergency department (ED) from June 2020 to March 2021. We conducted the study at the Children’s Hospital of San Antonio (CHofSA), a freestanding, 200-bed, tertiary care children’s hospital. The Baylor College of Medicine Institutional Review Board and CHofSA feasibility committee approved the study. Due to the prospective observational nature of the study, a waiver of informed consent was obtained.

In our study, we used ICON^®^ non-invasive hemodynamic monitor to measure objective hemodynamic parameters before and after FBs in children with shock in the ED. ICON^®^ monitor has been utilized to monitor patient hemodynamics in our pediatric intensive care unit (PICU) since 2018. Hence, we were interested to determine whether it may be feasible to expand the use of ICON^®^ monitor to the ED. After a short training period for the research team and ED staff, we began using the monitor on ED patients with shock to explore the workflow and obtain fluid resuscitation responses by measuring subjective and objective parameters.

### Inclusion Criteria

1. Children aged 0–17 years who presented in shock or presumed shock and required FB in our ED.
2. Children in whom all the hemodynamic parameters were available and feasible using ICON^®^ monitor.

### Exclusion Criteria

1. Children with suspected infection with SARS CoV-2 who were designated as patients under investigation (PUI).
2. Children in whom any hemodynamic data using ICON^®^ monitor were not feasible or not available.

Unfortunately, during the study period, we were facing the COVID-19 pandemic wave. During the study period, our hospital policies required all PUI to be considered as suspected COVID 19 and did not allow trainees to be involved with the care of PUI and patients with COVID-19. We therefore excluded the PUI.

We defined shock *a priori* based on a combination of clinical and/or laboratory parameters such as hypothermia or hyperthermia, tachycardia or tachycardia out of proportion to the degree of fever, tachypnea, hypotension, delayed capillary refill time, dry mucosa, and elevated lactate levels which warranted fluid resuscitation. In our ED, it is a standard protocol for the triage nurse to alert the ED attending about any patient presenting in shock or presumed shock. The ED attending then decides about the administration of FB based on his/her assessment of the patient’s clinical status suggestive of shock or presumed shock. Therefore, in our study, we included pediatric patients presenting with shock or presumed shock who were administered FB. In our ED, it is a routine practice to monitor the patients’ clinical and hemodynamic parameters obtained *via* standard monitoring before and after FB interventions. We expanded the current standard of care with an application of the ICON^®^ monitor to obtain additional hemodynamic parameters after the decision to administer fluid was made. The providers were blinded to the information collected by the monitor to reduce the risk of introducing bias into the treatment of the patients. We did not initiate or change real-time clinical management of the patient based on hemodynamic parameters obtained using the ICON^®^ monitor.

### Non-invasive Monitoring

**FIGURE 1:**
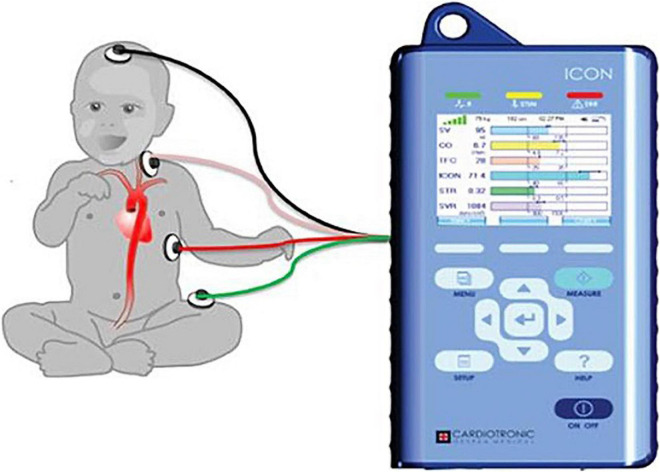
The figure shows placement of ICON four sensors. One sensor placed on forehead, second sensor placed on left base of neck, third sensor placed on left thorax at the level of xiphoid, and fourth sensor placed on left thigh. Adopted from ICON user manual with due permission from Markus Osypka, Osypka Medical Inc., Germany.

ICON^®^ monitor measurements require the placement of four skin sensors on the left side of the body as shown in Figure 1. After attaching the four sensors (two upper and two lower), we confirmed 100% signal strength before capturing hemodynamic parameters for accuracy. The upper sensors apply a harmless low amplitude, high-frequency alternating current. The changes in pulsatile red blood cell flow and change of thoracic electrical bio-impedance during the cardiac cycle are captured between the pair of the sensors. The complex mathematical algorithm built into the device calculates beat-to-beat parameters such as cardiac output, stroke volume, and many other hemodynamic parameters (25).

### Hemodynamic Measurements

We collected hemodynamic variables before and within 10 min after each FB in children with shock. We obtained demographic characteristics such as age and gender, clinical parameters such as heart rate (HR), respiratory rate (RR), systolic blood pressure (SBP), diastolic blood pressure (DBP), mean arterial pressure (MAP), and hemodynamic parameters from ICON device such as cardiac output (CO), cardiac index (CI), index of contractility (ICON), stroke volume (SV), stroke index (SI), corrected flow time (FTC), systolic time ratio (STR), variation of index of contractility (VIC), stroke volume variation (SVV), systemic vascular resistance (SVR), and thoracic fluid content (TFC).

Similar to the prior studies in adults, we defined the fluid responders *a priori* as those who exhibited an increase in SV by ≥10% after a single FB of 20 ml/kg crystalloid (18).

### Statistical Analysis

We conducted a statistical analysis using R–project (R Core Team, Vienna, Austria). We presented numeric data in median interquartile range (IQR) values. We calculated percent change (Δ) for pre- and post-hemodynamic data and compared the data using paired-sample Student’s *t*-test. We used Mann–Whitney *U*-test to compare hemodynamic parameters between fluid responders and non-responders. Furthermore, we plotted sensitivity and specificity for the cut-off points 5, 10, 15, and 20% for all variables to create receiver operating characteristic (ROC) curves to assess their predictive performances. *p*-values < 0.05 were considered statistically significant.

## Results

**FIGURE 2:**
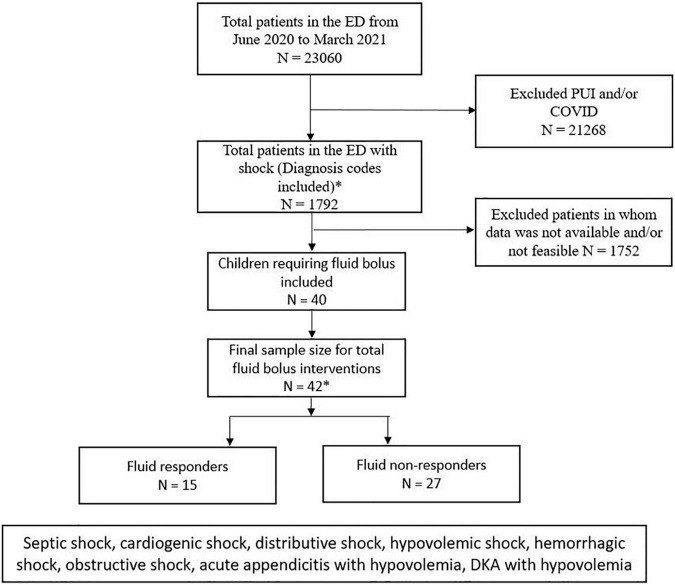
Consort patient flow diagram showing the total number of ED visits in our study period and number of patients who were included and excluded in the study. PUI, patients under investigation, COVID, coronavirus disease.

During the study period between June 2020 and March 2021, total number of ED visits in our children’s hospital was 23,060. Out of these patients, 1,792 presented with shock. During the Coronavirus disease 2019 (COVID-19) pandemic, the ICON device was not used in many patients who visited our ED during the study period. Therefore, in total, we were able to record 42 fluid interventions in 40 patients out of these 1,792 during our pilot study (Figure 2).

### Patient’s Characteristics

The median (IQR) patient age was 10.56 (4.8, 14.8) years with male/female ratio 1.2:1. Out of 40 patients included in our study, 32 (80%) had clinical findings and the remaining 8 (20%) had clinical and laboratory findings suggestive of shock. The hypovolemic shock was present to some degrees in 72% and septic shock in 28% of patients. The most common underlying etiology of shock was hypovolemia due to gastrointestinal condition (48%) (Table 1). All patients received FBs with crystalloids *via* infusion pump. The preferred crystalloid was normal saline in 32/42 (76%) and ringer’s lactate in 10/42 (24%). The mean weight-based volume of fluid administration was 16.24 ml/kg (standard deviation 5.6) with a mean duration of 47.26 min (standard deviation 15.8) to bolus completion.

**TABLE 1:** Demographic data and clinical characteristics.

Patient’s characteristics	Percentage (%) or Median (IQR)
Age (years)	10.56 (4.8, 14.8)
Gender (male: female)	1.2:1
**Type of shock**	
Hypovolemic	30 (72%)
Septic	12 (28%)
**Underlying etiology/Primary diagnosis/System involved**	
Gastrointestinal	20 (48%)
Endocrine/Metabolic	9 (22%)
Neurological	7 (16%)
Genitourinary	2 (6%)
Respiratory	1 (2%)
Musculoskeletal	1 (2%)
Hematology/Oncology	2 (4%)

*IQR, interquartile range.*

### Effects of Fluid Therapy and Fluid Responsiveness

We observed a significant decrease in ΔRR [−1.61 (−14.8, 0); *p* = 0.012], ΔDBP [−5.5 (−14.4, 8); *p* = 0.027], ΔMAP [−2.2 (−11, 2); *p* = 0.018], ΔSVR [−5.8 (−20, 5.2); *p* = 0.025], and ΔSTR [−8.39 (−21, 3); *p* = 0.001] and a significant increase in ΔTFC [6.2 (3.5, 11.4); *p* = 0.01] following FB. About 35% of FBs led to > / = 10% change in TFC. There were no significant differences in HR, SBP, SV, SI, CO, CI, SVV, FTC, ICON, and VIC post-FB (Table 2).

**TABLE 2:** Comparison between pre- and post-FB hemodynamic variables.

Parameters	Pre-FB median (IQR)	Post-FB median (IQR)	Δ Median (IQR)	*p*-values
HR (bpm)	107 (92, 131)	103.5 (85, 127)	−0.72 (−8, 4.2)	0.453
RR (/min)	22 (19, 24)	20 (18, 24)	−1.61 (−14.8, 0)	**0.012**
SBP (mmHg)	116 (105, 125)	112.5 (100, 123)	−5 (−14.5, 7)	0.104
DBP (mmHg)	73 (64, 84)	69 (55, 80)	−5.5 (−14.4, 8)	**0.027**
MAP (mmHg)	88 (83, 96)	84.5 (73, 93)	−2.2 (−11, 2)	**0.018**
SV (ml)	51.5 (27, 77)	54.5 (35, 72)	0.7 (−9, 15)	0.814
SI (BSA)	38 (35, 48)	41 (34, 48)	2.16 (−7.5, 16)	0.242
CO (l/min)	5 (3.5, 7.3)	4.7 (3.8, 6.3)	2.4 (−8.4, 16)	0.858
CI (BSA)	4.2 (3.2, 4.8)	4.2 (3.3, 5)	1.1 (−8, 16)	0.2577
SVV (%)	12.5 (8, 17)	13 (8, 17)	0.0 (−5, 4)	0.509
FTC (ms)	312 (298, 327)	323.5 (302, 333)	2.31 (−0.8, 8)	0.282
TFC	28.5 (20, 34)	31 (23, 37)	6.2 (3.5, 11.4)	**0.005**
SVR (dyn.s/cm^5)^	1387 (958, 1913)	1374 (942, 1704)	−5.8 (−20, 5.2)	**0.025**
STR	0.37 (0.33, 0.45)	0.34 (0.3, 0.38)	−8.39 (−21, 3)	**0.001**
ICON	72.8 (56.6, 96.3)	71 (51, 103)	4 (−21.6, 16.6)	0.858
VIC (%)	13.5 (9, 27)	16.5 (11, 26)	0.0 (−33, 57)	0.433

*FB, fluid bolus; HR, heart rate; RR, respiratory rate; SBP, systolic blood pressure; DBP, diastolic blood pressure; MAP, mean arterial pressure; SV, stroke volume; SI, stroke index; CO, cardiac output; CI, cardiac index; SVV, stroke volume variation; FTC, corrected flow time; TFC, thoracic fluid content; SVR, systemic vascular resistance; STR, systolic time ratio; ICON, index of contractility; VIC, variation of index of contractility; Δ, percentage change; bpm, beats per minute;/min = per minute; ml, milliliters; BSA, body surface area; %, percentage; ms, milliseconds; dyn.s/cm^5^, dynes/sec/cm^5^; significant value, p < 0.05. (Paired-sample Student’s t-test). Bold values correspond to significant p-values.*

Fluid responsiveness was seen in 15/42 interventions. Between responders vs. non-responders, fluid responders had a significant decrease in ΔHR [−3.7 (−16.6, −0.72) vs. 1.32 (−5.1, 9.9); *p* = 0.002], ΔSVV [−34 (−50, −2.9) vs. 25 (−19.5, 83.7); *p* = 0.002], ΔSVR [−12.8 (−22.7, −7.7) vs. 0 (−12.8, 9.2); *p* = 0.31], and ΔSTR [−19.3 (−24.6, −12) vs. −5 (−14.8, 8.8); *p* = 0.03]. Furthermore, fluid responders had a significant increase in ΔSI [16 (15.8, 24.2) vs. −3 (−9, 0.91); *p* = 0.00000011], ΔCO [14.2 (4, 23) vs. −2.2 (−12.9, 6.2); *p* = 0.006], ΔCI [14.5 (5.2, 23) vs. −3.5 (−12.8, 5.6); *p* = 0.003], ΔFTC [7.65 (2.6, 9.4) vs. 0 (−6, 3.8); *p* = 0.002], and ΔICON [16.7 (13.5, 25) vs. −9 (−23, 5.1), *p* = 0.003] as compared to non-responders. There were no significant differences between age, ΔRR, ΔSBP, ΔDBP, ΔMAP, ΔTFC, and ΔVIC between these two groups (Table 3).

**TABLE 3:** Changes in hemodynamic variables in responders vs. non-responders.

Parameters	Responders *n* = 15 median (IQR)	Non-responders *n* = 27 median (IQR)	*p*-values
Age (years)	7.9 (2, 11)	12.3 (5.6, 15.6)	0.15
ΔHR (bpm)	−3.7 (−16.6, −0.72)	1.32 (−5.1, 9.9)	**0.002**
ΔRR (/min)	−6.2 (−15.5, 0)	0 (−12.7, 4.5)	0.169
ΔSBP (mmHg)	−0.85 (−4.9, 4.9)	−6.6 (−12, 7.5)	0.293
ΔDBP (mmHg)	−2.4 (−5.8, 8.9)	−9.8 (−16.2, 6.43)	0.253
ΔMAP (mmHg)	−1,1 (−4.6, 1.8)	−6.5 (−12.8, −0.6)	0.100
ΔSI (BSA)	16 (15.8, 24.2)	−3 (−9, 0.91)	**0.00000011**
ΔCO (l/min)	14.2 (4, 23)	−2.2 (−12.9, 6.2)	**0.006**
ΔCI (BSA)	14.5 (5.2, 23)	−3.5 (−12.8, 5.6)	**0.003**
ΔSVV (%)	−34 (−50, −2.9)	25 (−19.5, 83.7)	**0.002**
ΔFTC (ms)	7.65 (2.6, 9.4)	0 (−6, 3.8)	**0.002**
ΔTFC	6.6 (5.4, 12)	5.8 (1.5, 10.5)	0.216
ΔSVR (dyn.s/cm^5^)	−12.8 (−22.7, −7.7)	0 (−12.8, 9.2)	**0.031**
ΔSTR	−19.3 (−24.6, −12)	−5 (−14.8, 8.8)	**0.003**
ΔICON	16.7 (13.5, 25)	−9 (−23, 5.1)	**0.0003**
ΔVIC (%)	0 (−39, 44)	0 (−23, 71)	0.617

*HR, heart rate; RR, respiratory rate; SBP, systolic blood pressure; DBP, diastolic blood pressure; MAP, mean arterial pressure; SV, stroke volume; SI, stroke index; CO, cardiac output; CI, cardiac index; SVV, stroke volume variation; FTC, corrected flow time; TFC, thoracic fluid content; SVR, systemic vascular resistance; STR, systolic time ratio; ICON, index of contractility; VIC, variation of index of contractility; Δ, percentage change; bpm = beats per minute;/min = per minute; millimeters of mercury, mmHg; ml, milliliters; BSA, body surface area; %, percentage; ms, milliseconds; dyn.s/cm^5^, dynes/sec/cm^5^; significant value, p < 0.05. Bold values correspond to significant p-values.*

### Receiver Operating Characteristic Curve Analysis

**FIGURE 3:**
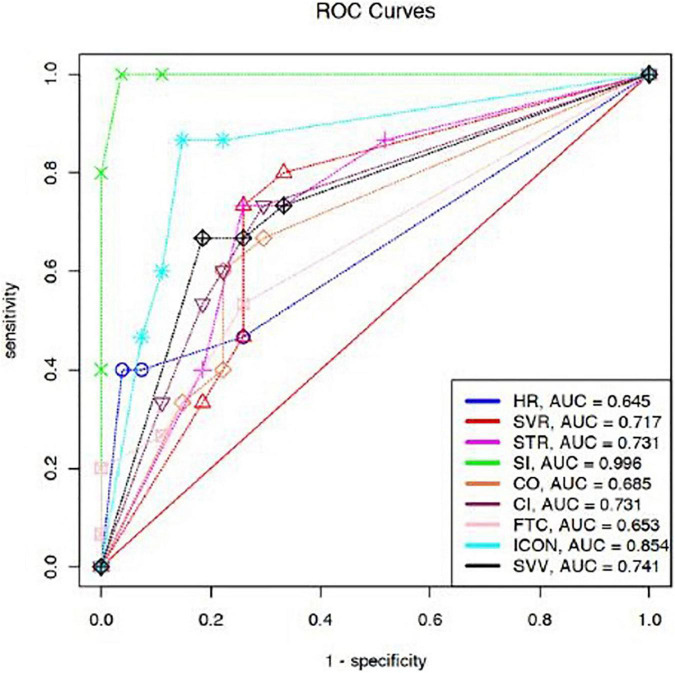
Comparison of AUCs for prediction of FR after fluid expansion. ΔHR [0.64 (95% CI 0.55–0.72)], ΔSVR [0.71 (95% CI 0.62–0.79)], ΔSVV [0.74 (95% CI 0.65–0.82)], ΔSTR [0.73 (95% CI 0.64–0.81)], ΔSI [0.99 (95% CI 0.97–1)], ΔCO [0.68 (95% CI 0.59–0.76)], ΔCI [0.73 (95% CI 0.64–0.81)], ΔFTC [0.65 (95% CI 0.56–0.73)], and ΔICON [0.85 (95% CI 0.78–0.91)]. HR, heart rate; RR, respiratory rate; SBP, systolic blood pressure; DBP, diastolic blood pressure; MAP, mean arterial pressure; SV, stroke volume; SI, stroke index; CO, cardiac output; CI, cardiac index; SVV, stroke volume variation; FTC, corrected flow time; TFC, thoracic fluid content; SVR, systemic vascular resistance; STR, systolic time ratio; ICON, index of contractility; VIC, variation of index of contractility; Δ, percentage change.

We compared areas under ROC curves (AUCs) after fluid expansion for ΔHR, ΔSVR, ΔSVV, ΔSTR, ΔSI, ΔCO, ΔCI, ΔFTC, and ΔICON. The AUC denoted better classifiers for ΔSI (AUC–0.99), ΔICON (AUC–0.85), and ΔCI (AUC–0.73). The optimal threshold value for CI, SI, and ICON calculated by the ROC curve analysis was 15% (Figure 3).

## Discussion

Our prospective observational pilot study focused on subjective and objective methods of assessing hemodynamic status and FR in children presenting to the ED in shock. To our knowledge, this is the first pediatric report of the use of electrical cardiography which demonstrated a significant change in objective indices of hemodynamic status following FB in children with shock. Additionally, the study demonstrated the changes in specific hemodynamic indices such as CI, SI, and ICON as measured by electrical cardiography best-predicted FR in children with shock. The overall goal of fluid administration in patients with shock is to increase cardiac preload and subsequently stroke volume (15, 17). However, the studies in critically ill patients that examined FR demonstrated only 40–50% of patients responded to volume expansion (19, 26, 27). Furthermore, the conventional subjective parameters such as vital signs and physical examination pose several limitations and alone are not reliable in assessing FR, especially in children (28, 29). On the other hand, objective parameters such as CO, CI, SV, SI, and SVR may provide much more accurate hemodynamic status (30). More recently, non-invasive cardiac output monitors are also being utilized to provide continuous data of patients’ hemodynamic status that could help in guiding fluid therapy and to predict FR (22, 31). Several studies have demonstrated the usefulness of these non-invasive devices in adults (23, 24, 32), yet the effectiveness of such devices remains controversial in pediatric patients. Some studies support the use of these newer non-invasive techniques in children. A study by Norozi et al. demonstrated a good correlation (*r* = 0.84) between CO measurements obtained by non-invasive cardiac output monitor and invasive direct Fick oxygen method in children with various congenital heart conditions (33). However, in another study that compares SVV measured by NICOM (non-invasive cardiac output monitor), traditional transesophageal echocardiography reported its ineffectiveness to predict FR in children undergoing cardiac surgery (34). In another study by Ballestero Yolanda et al. on a pediatric animal model with hemorrhagic shock, CI measured by bioreactance technique did not show significant changes after volume expansion (35).

In our study, we observed a significant decrease in RR, MAP, SVR, and STR and an increase in TFC, while CO, CI, SV, and SI remained unchanged. TFC is a newer objective parameter that has shown to identify pulmonary congestion which might not be evident on chest x-rays (36, 37). One study in critically ill children with respiratory failure and shock demonstrated that high TFC values correlate with pulmonary plethora and predicted patient outcomes (37). Another study in patients with heart failure suggested that TFC may identify patients at risk for decompensation (38). TFC measurements using EC have shown to be correlated with the presence of respiratory distress in infants (39). Hence, our pilot study that determined a significant increase in TFC may identify this as an important area of future study. A significant increase in TFC following FB in our study may be related to increased pulmonary interstitial edema following FB.

Predicting FR in children can be challenging. The predictive abilities of various hemodynamic parameters have previously been evaluated in a systematic review (40). Our results show that ΔCI, ΔSI, and ΔICON had good predictability for FR in children. In our study, AUCs of CI, SI, and ICON were 0.73, 0.99, and 0.85, respectively. In a study on adult patients undergoing laparoscopic cholecystectomy, AUCs for CI and SI for FR were 0.83 and 0.90, respectively (41). In a study on post-operative pediatric patients, AUC for SI was 0.88 (42). Our findings of AUC for HR for FR correlate with the prior similar results (43, 44). Though commonly used as a parameter to assess fluid status, HR is a worse predictor of FR. In our study, changes in CI and ICON showed a high predictive performance for FR. It is possible that FB improved myocardial contractility through Frank–Starling mechanism, and therefore, CI and ICON showed an excellent predictive performance.

### Limitations

Our study had several limitations. It is a prospective observational pilot study conducted in a single center, and our sample size is small. A major challenge we encountered that led to a smaller sample size was the necessary exclusion of patients with COVID-19 or PUI during the unprecedented pandemic due to PPE restrictions and institutional guidelines to protect students from potential exposure. In addition, this cohort does not represent consecutive patients due to the limitations of availability and feasibility to perform monitoring in the unpredictable ED setting. Furthermore, considering these limitations in patient recruitment, our sample included most patients with mild-to-moderate shock and may not be representative of the most critically ill patients. Additionally, this may have impacted the rate of fluid administration with the mean bolus duration longer than optimum for shock resuscitation guidelines. Therefore, the results of this study cannot be generalized to all patients with pediatrics with shock. Despite the limitations of our pilot, the parameters we found to be statistically significant demonstrate the promising results that using a non-invasive monitor to assess objective hemodynamic changes in children with shock has the potential to aid ED physicians in predicting FR and better guide fluid management.

## Conclusion

The results of our pilot study suggest that integration of objective assessment with subjective data using advanced non-invasive monitoring could help to evaluate patients’ hemodynamic status and FR in children with shock in ED settings. The findings of excellent predictive performance (AUC 0.85) of changes in ICON for FR could potentially aid treating physicians in avoiding fluid overload, developing optimal management plans, and using objective clinical decision-making for children with shock. Larger, multi-center, prospective, randomized studies are needed to further evaluate the validity of non-invasive devices in predicting FR in children.

## Data Availability Statement

The raw data supporting the conclusions of this article will be made available by the authors, without undue reservation.

## Ethics Statement

The studies involving human participants were reviewed and approved by the Baylor College of Medicine IRB. Written informed consent from the participants’ legal guardian/next of kin was not required to participate in this study in accordance with the national legislation and the institutional requirements.

## Author Contributions

All authors listed have made a substantial, direct, and intellectual contribution to the work, and approved it for publication.

## Conflict of Interest

The authors declare that the research was conducted in the absence of any commercial or financial relationships that could be construed as a potential conflict of interest.

## Publisher’s Note

All claims expressed in this article are solely those of the authors and do not necessarily represent those of their affiliated organizations, or those of the publisher, the editors and the reviewers. Any product that may be evaluated in this article, or claim that may be made by its manufacturer, is not guaranteed or endorsed by the publisher.

## References

[1] Mendelson J. Emergency department management of pediatric shock. *Emerg Med Clin North Am.* (2018) 36:427–40. 10.1016/j.emc.2017.12.010 29622332

[2] Martin K Weiss SL. Initial resuscitation and management of pediatric septic shock. *Minerva Pediatric.* (2015) 67:141–58. PMC4395852 25604591

[3] de Souza DC Shieh HH Barreiro ER Ventura AM Bousso A Troster EJ Epidemiology of sepsis in children admitted to PICUs in South America. *Pediatric Crit Care Med J Soc Crit Care Med World Federation Pediatric Intens Crit Care Soc.* (2016) 17:727–34. 10.1097/PCC.0000000000000847 27362850

[4] Weiss SL Fitzgerald JC Pappachan J Wheeler D Jaramillo-Bustamante JC Salloo A Global epidemiology of pediatric severe sepsis: the sepsis prevalence, outcomes, and therapies study. *Am J Respirat Crit Care Med.* (2015) 191:1147–57. 10.1164/rccm.201412-2323OC 25734408 PMC4451622

[5] Carcillo JA Han K Lin J Orr R. Goal-directed management of pediatric shock in the emergency department. *Clin Pediatric Emerg Med.* (2007) 8:165–75. 10.1016/j.cpem.2007.07.002

[6] Davis AL Carcillo JA Aneja RK Deymann AJ Lin JC Nguyen TC American college of critical care medicine clinical practice parameters for hemodynamic support of pediatric and neonatal septic shock. *Crit Care Med.* (2017) 45:1061–93. 10.1097/CCM.0000000000002425 28509730

[7] Levy MM Evans LE Rhodes A. The surviving sepsis campaign bundle: 2018 update. *Intens Care Med.* (2018) 44:925–8. 10.1007/s00134-018-5085-0 29675566

[8] Lee SJ Ramar K Park JG Gajic O Li G Kashyap R. Increased fluid administration in the first three hours of sepsis resuscitation is associated with reduced mortality: a retrospective cohort study. *Chest.* (2014) 146:908–15. 10.1378/chest.13-2702 24853382 PMC4188147

[9] Glassford NJ Eastwood GM Bellomo R. Physiological changes after fluid bolus therapy in sepsis: a systematic review of contemporary data. *Crit Care (London, England).* (2014) 18:696. 10.1186/s13054-014-0696-5 25673138 PMC4331149

[10] Boyd JH Forbes J Nakada TA Walley KR Russell JA. Fluid resuscitation in septic shock: a positive fluid balance and elevated central venous pressure are associated with increased mortality. *Crit Care Med.* (2011) 39:259–65. 10.1097/CCM.0b013e3181feeb15 20975548

[11] Lopes C Piva JP. Fluid overload in children undergoing mechanical ventilation. Sobrecarga hídrica em crianças submetidas à ventilação mecânica. *Revista Brasileira Terapia Intensiva.* (2017) 29:346–53. 10.5935/0103-507X.20170045 28977099 PMC5632978

[12] Micek ST McEvoy C McKenzie M Hampton N Doherty JA Kollef MH. Fluid balance and cardiac function in septic shock as predictors of hospital mortality. *Crit Care (London, England).* (2013) 17:R246. 10.1186/cc13072 24138869 PMC4056694

[13] Maitland K Kiguli S Opoka RO Engoru C Olupot-Olupot P Akech SO Mortality after fluid bolus in African children with severe infection. *N Engl J Med.* (2011) 364:2483–95. 10.1056/NEJMoa1101549 21615299

[14] Carcillo JA Davis AL Zaritsky A. Role of early fluid resuscitation in pediatric septic shock. *JAMA.* (1991) 266:1242–5. 10.1001/jama.1991.03470090076035 1870250

[15] Gelbart B. Fluid bolus therapy in pediatric sepsis: current knowledge and future direction. *Front Pediatrics.* (2018) 6:308. 10.3389/fped.2018.00308 30410875 PMC6209667

[16] Hjortrup PB Haase N Bundgaard H Thomsen SL Winding R Pettilä V Restricting volumes of resuscitation fluid in adults with septic shock after initial management: the CLASSIC randomised, parallel-group, multicentre feasibility trial. *Intens Care Med.* (2016) 42:1695–705. 10.1007/s00134-016-4500-7 27686349

[17] Parker MJ Thabane L Fox-Robichaud A Liaw P Choong K Canadian Critical Care Trials Group and the Canadian Critical Care Translational Biology Group. A trial to determine whether septic shock-reversal is quicker in pediatric patients randomized to an early goal-directed fluid-sparing strategy versus usual care (SQUEEZE): study protocol for a pilot randomized controlled trial. *Trials.* (2016) 17:556. 10.1186/s13063-016-1689-2 27876084 PMC5120449

[18] Marik PE Monnet X Teboul JL. Hemodynamic parameters to guide fluid therapy. *Ann Intens Care.* (2011) 1:1. 10.1186/2110-5820-1-1 21906322 PMC3159904

[19] Mackenzie DC Noble VE. Assessing volume status and fluid responsiveness in the emergency department. *Clin Exp Emerg Med.* (2014) 1:67–77. 10.15441/ceem.14.040 27752556 PMC5052829

[20] Latham HE Bengtson CD Satterwhite L Stites M Subramaniam DP Chen GJ Stroke volume guided resuscitation in severe sepsis and septic shock improves outcomes. *J Crit Care.* (2017) 42:42–6. 10.1016/j.jcrc.2017.06.028 28672146

[21] Douglas IS Alapat PM Corl KA. Fluid response evaluation in sepsis hypotension and shock: a randomized clinical trial. *Chest.* (2020) 158:1431–45. 10.1016/j.chest.2020.04.025 32353418 PMC9490557

[22] Nowak RM Nanayakkara P DiSomma S Levy P Schrijver E Huyghe R Noninvasive hemodynamic monitoring in emergency patients with suspected heart failure, sepsis and stroke: the PREMIUM registry. *Western J Emerg Med.* (2014) 15:786–94. 10.5811/westjem.2014.8.21357 25493119 PMC4251220

[23] Zoremba N Bickenbach J Krauss B Rossaint R Kuhlen R Schälte G. Comparison of electrical velocimetry and thermodilution techniques for the measurement of cardiac output. *Acta Anaesthesiol Scand.* (2007) 51:1314–9. 10.1111/j.1399-6576.2007.01445.x 17944633

[24] Kuster M Haltmeier T Exadaktylos A Schnüriger B. Non-invasive cardiac output monitoring device “ICON” in trauma patients: a feasibility study. *Eur J Trauma Emerg Surg Off Publ Eur Trauma Soc.* (2019) 45:1069–76. 10.1007/s00068-018-0984-x 30014271

[25] Sumbel L Annamalai MR Wats A Salameh M Agarwal A Bhalala U. Noninvasive cardiac output monitoring using electrical cardiometry and outcomes in critically ill children. *J Pediatric Intens Care.* (2020): 10.1055/s-0040-1718867 PMC9208845 35734208

[26] Marik PE Cavallazzi R Vasu T Hirani A. Dynamic changes in arterial waveform derived variables and fluid responsiveness in mechanically ventilated patients: a systematic review of the literature. *Crit Care Med.* (2009) 37:2642–7. 10.1097/CCM.0b013e3181a590da 19602972

[27] Michard F Teboul JL. Predicting fluid responsiveness in ICU patients: a critical analysis of the evidence. *Chest.* (2002) 121:2000–8. 10.1378/chest.121.6.2000 12065368

[28] Pereira de Souza Neto E Grousson S Duflo F Ducreux C Joly H Convert J Predicting fluid responsiveness in mechanically ventilated children under general anaesthesia using dynamic parameters and transthoracic echocardiography. *Br J Anaesthesia.* (2011) 106:856–64. 10.1093/bja/aer090 21525016

[29] Kluckow M Evans N. Relationship between blood pressure and cardiac output in preterm infants requiring mechanical ventilation. *J Pediatrics.* (1996) 129:506–12. 10.1016/s0022-3476(96)70114-2 8859256

[30] O’Neill R Dempsey EM Garvey AA Schwarz CE. Non-invasive cardiac output monitoring in neonates. *Front Pediatrics.* (2021) 8:614585. 10.3389/fped.2020.614585 33585366 PMC7880199

[31] Noori S Drabu B Soleymani S Seri I. Continuous non-invasive cardiac output measurements in the neonate by electrical velocimetry: a comparison with echocardiography. *Arch Dis Child Fetal Neonatal Ed.* (2012) 97:F340–3. 10.1136/fetalneonatal-2011-301090 22933092

[32] Marik PE Levitov A Young A Andrews L. The use of bioreactance and carotid Doppler to determine volume responsiveness and blood flow redistribution following passive leg raising in hemodynamically unstable patients. *Chest.* (2013) 143:364–70. 10.1378/chest.12-1274 22910834

[33] Norozi K Beck C Osthaus WA Wille I Wessel A Bertram H. Electrical velocimetry for measuring cardiac output in children with congenital heart disease. *Br J Anaesthesia.* (2008) 100:88–94. 10.1093/bja/aem320 18024954

[34] Lee JH No HJ Song IK Kim HS Kim CS Kim JT. Prediction of fluid responsiveness using a non-invasive cardiac output monitor in children undergoing cardiac surgery. *Br J Anaesthesia.* (2015) 115:38–44. 10.1093/bja/aev109 25926311

[35] Ballestero Y Urbano J López-Herce J Solana MJ Botrán M Vinciguerra D Pulmonary arterial thermodilution, femoral arterial thermodilution and bioreactance cardiac output monitoring in a pediatric hemorrhagic hypovolemic shock model. *Resuscitation.* (2012) 83:125–9. 10.1016/j.resuscitation.2011.06.039 21763249

[36] Sumbel L Wats A Salameh M Appachi E Bhalala U. Thoracic Fluid Content (TFC) measurement using impedance cardiography predicts outcomes in critically ill children. *Front Pediatrics.* (2021) 8:564902. 10.3389/fped.2020.564902 33718292 PMC7947197

[37] Fathy S Hasanin AM Raafat M Mostafa MMA Fetouh AM Elsayed M Thoracic fluid content: a novel parameter for predicting failed weaning from mechanical ventilation. *J Intens Care.* (2020) 8:20. 10.1186/s40560-020-00439-2 32161651 PMC7059362

[38] Folan L Funk M. Measurement of thoracic fluid content in heart failure: the role of impedance cardiography. *AACN Adv Crit Care.* (2008) 19:47–55. 10.1097/01.AACN.0000310751.93287.42 18418105

[39] Paviotti G De Cunto A Moressa V Bettiol C Demarini S. Thoracic fluid content by electric bioimpedance correlates with respiratory distress in newborns. *J Perinatol Off J California Perinatal Assoc.* (2017) 37:1024–7. 10.1038/jp.2017.100 28749485

[40] Gan H Cannesson M Chandler JR Ansermino JM. Predicting fluid responsiveness in children: a systematic review. *Anesthesia Analgesia.* (2013) 117:1380–92. 10.1213/ANE.0b013e3182a9557e 24257389

[41] Moon EJ Lee S Yi JW Kim JH Lee BJ Seo H. Stroke volume variation and stroke volume index can predict fluid responsiveness after mini-volume challenge test in patients undergoing laparoscopic cholecystectomy. *Medicina (Kaunas, Lithuania).* (2019) 56:3. 10.3390/medicina56010003 31861707 PMC7022270

[42] Vergnaud E Vidal C Verchère J Miatello J Meyer P Carli P Stroke volume variation and indexed stroke volume measured using bioreactance predict fluid responsiveness in postoperative children. *Br J Anaesthesia.* (2015) 114:103–9. 10.1093/bja/aeu361 25315146

[43] Byon HJ Lim CW Lee JH Park YH Kim HS Kim CS Prediction of fluid responsiveness in mechanically ventilated children undergoing neurosurgery. *Br J Anaesthesia.* (2013) 110:586–91. 10.1093/bja/aes467 23250892

[44] Renner J Broch O Gruenewald M Scheewe J Francksen H Jung O Non-invasive prediction of fluid responsiveness in infants using pleth variability index. *Anaesthesia.* (2011) 66:582–9. 10.1111/j.1365-2044.2011.06715.x 21539529

